# The Effect of High-Temperature Heating on Amounts of Bioactive Compounds and Antiradical Properties of Refined Rapeseed Oil Blended with Rapeseed, Coriander and Apricot Cold-Pressed Oils

**DOI:** 10.3390/foods13152336

**Published:** 2024-07-25

**Authors:** Monika Fedko, Aleksander Siger, Aleksandra Szydłowska-Czerniak, Dobrochna Rabiej-Kozioł, Alicja Tymczewska, Katarzyna Włodarczyk, Dominik Kmiecik

**Affiliations:** 1 Department of Food Technology and Assessment, Institute of Food Science, Warsaw University of Life Sciences, Nowoursynowska 159c, 02-787 Warsaw, Poland; 2 Department of Food Biochemistry and Analysis, Poznań University of Life Sciences, Wojska Polskiego 31, 60-634 Poznań, Poland; aleksander.siger@up.poznan.pl; 3 Department of Analytical Chemistry and Applied Spectroscopy, Faculty of Chemistry, Nicolaus Copernicus University in Toruń, Gagarina 7, 87-100 Toruń, Poland; olasz@umk.pl (A.S.-C.); d.rabiej@umk.pl (D.R.-K.); alicjatymczewska@gmail.com (A.T.); katarzyna.wlodarczyk25@gmail.com (K.W.); 4 Department of Food Technology of Plant Origin, Poznań University of Life Sciences, Wojska Polskiego 31, 60-634 Poznań, Poland; dominik.kmiecik@up.poznan.pl

**Keywords:** phytosterols, tocochromanols, tocopherols, radical scavenging activity, DPPH, ABTS, frying, rapeseed oil, coriander seed oil, apricot kernel oil

## Abstract

Cold-pressed oils are rich sources of bioactive substances, which may protect triacylglycerols from degradation during frying. Nevertheless, these substances may decompose under high temperature. This work considers the content of bioactive substances in blends and their changes during high-temperature heating. Blends of refined rapeseed oil with 5% or 25% in one of three cold-pressed oils (rapeseed, coriander and apricot) were heated at 170 or 200 °C in a thin layer on a pan. All non-heated blends and cold-pressed oils were tested for fatty acid profile, content and composition of phytosterols, tocochromanols, chlorophyll and radical scavenging activity (RSA) analyzed by 2,2-diphenyl-1-picrylhydrazyl (DPPH), and 2,2′-azino-bis(3-ethylbenzothiazoline-6-sulphonic acid) (ABTS) assays. Moreover, the stability of phytosterols, tocochromanols, DPPH and ABTS values was determined in heated blends. All tocochromanols were lost during the heating process, in particular, at 200 °C. However, there were some differences between homologues. α-Tocopherol and δ-tocopherol were the most thermolabile and the most stable, respectively. Phytosterols were characterized by very high stability at both temperatures. We observed relationships between ABTS and DPPH values and contents of total tocochromanols and α-tocopherol. The obtained results may be useful in designing a new type of fried food with improved health properties and it may be the basis for further research on this topic.

## 1. Introduction

Pan frying allows to obtain dishes desirable by consumers due to their unique taste, rich aroma, golden colour and crispy texture [1]. It is a budget-friendly and fast process. However, conditions applied during frying may induce a cascade of adverse degradation oil reactions. The exposition of oil to the oxygen from the air initiates oxidation reaction, which is defined as autoxidation. It is a free radical chain reaction, which may be catalyzed by different factors including heat, light, metal ions and whose main products are hydroperoxides (lipid oxidation’s primary products). Under high temperatures during frying, hydroperoxides are decomposed into more advanced degradation products (lipid oxidation’s secondary products) which are attributed to thermal oxidation [2,3]. Products such as polar compounds and triacylglycerol (TAG) polymers are generated, which may contribute to a lot of healthy problems, including obesity, diabetes, cardiovascular diseases and cancer [1,4,5,6], and thus, improving the thermal stability of oils is an important scientific issue.

The synthetic antioxidants supplemented into oils may limit their degradation during frying. Nevertheless, these substances are suspected of being carcinogenic [7,8,9]. Previous experiments showed plant extracts, usually based on ethanol, introduced into the oil, may be efficient in protecting components of oils [10,11]. However, it is problematic due to the poor miscibility of oils with polar solvents, expensiveness and non-ecological procedures, which have unclear health effects. An interesting, relatively inexpensive and easy to apply solution is blending refined oil with cold-pressed oils in order to limit the deterioration of oils during frying.

Vegetable oils are composed of two fractions: saponifiable and unsaponifiable. The former shares approximately 98–99% of oil composition and consists mainly of TAG fatty acids. The latter is about 1–2% of oil composition and phytosterols, and tocochromanols constitute the bulk of the unsaponifiable lipid fraction [12]. Beneficial effects on the health of tocochromanols and phytosterols have been demonstrated in numerous studies [13,14,15,16]. Moreover, endogenous, bioactive substances found in oils may limit TAG degradation during frying, including oxidation and polymerization [17,18,19]. On the other hand, these substances are characterized by varied, often high lability under high-temperature conditions used during thermal treatment [20,21]. Oils obtained from different raw materials may have different compositions of both fatty acids and bioactive substances [12,22,23,24] and thus differ in their stability under the influence of high temperature. In turn, the properties of individual types of bioactive substances depend mainly on their structure, but also on the environment, the occurrence of synergistic or antagonistic interactions and other interactions with the matrix. Blends with increased thermal resistance should be based on oils with a high proportion of monounsaturated fatty acids (MUFA). This is due to their lower susceptibility to thermal degradation compared to polyunsaturated fatty acids [25,26] and, on the other hand, to their beneficial effect on health, including the cardiovascular system, compared to saturated fatty acids [27,28,29]. In our geographic region, there are only a few kinds of oils with a high proportion of MUFA, including rapeseed oil, coriander seed oil and apricot oil. Numerous publications have shown that they are a rich source of bioactive, antioxidant substances [30,31,32,33,34]. Coriander seed oil is distinguished by the presence of tocotrienols, which, according to some sources, have better antioxidant properties than tocopherols [35,36]. Apricot seed oil is obtained from a product of the food industry that is difficult to fully utilize, so it fits into the current pro-ecological policy.

Most of the current works of blends with thermal stability focus on the analysis of degradation products and contain only limited data on bioactive substances and changes in their content [37,38,39]. Moreover, the effect of individual bioactive substances content on the antioxidant activity of oils and their interactions with one another is still unclear. For example, according to some authors, the in vitro antioxidant activity of individual tocopherol homologues is in the order of δ-T > γ-T ≈ ß-T > α-T [15]. Other authors reported that α-tocopherol shows the highest antioxidant activity [40,41]. Athanasiadis et al. [42] presented the antioxidant capacity of tocopherols, which ranked in the following order: δ > γ > α > β at temperatures from 80 to 120 °C and in the order of α > γ > β > δ at temperatures between 20 and 60 °C. Under frying conditions (160 °C), γ- and δ-tocotrienols were significantly more active than their corresponding tocopherol homologs [43]. This effect was not observed for α-tocopherol and α-tocotrienols. Interestingly, δ-tocotrienol activity increased in higher concentrations, but γ-tocotrienol did not depend on concentration. Moreover, at the lower temperature (60 °C), α- and β-tocotrienols reduced coconut oil stability and showed pro-oxidative effects. Müller et al. [44] stated that the relative effectiveness of tocochromanols depends on the experimental conditions. Some investigators have reported the antioxidant or anti-polymerization activity of phytosterols, attributed to the presence of an ethylene group in their side-chain, such as Δ^5^- and Δ^7^-avenasterol and citrostadienol [45,46] or co-occurrence of an ethylidene group in the side chain and two endocyclic double bonds at C-8 and C-14 like in vernosterol. Other authors [47] discussed the pro-oxidant effect of phytosterols on the oxidation of encapsulated emulsions. Interesting results were obtained by Chang et al. [48], who indicated stigmasterol acted as a prooxidant at 60 °C, but as an antioxidant at 180 °C. A full understanding of these phenomena requires more research using various conditions, systems and materials.

To the best of our knowledge, there are no studies that would evaluate the effect of thermal treatment on the compositions and contents of tocochromanols, phytosterols and the radical scavenging activity (RSA) of refined rapeseed oil blended with cold-pressed rapeseed, coriander seed and apricot kernel oils in one publication

Therefore, this research aimed to improve the thermo-stability of refined rapeseed oil by blending it with cold-pressed rapeseed, coriander seed and apricot kernel oils. The effect of the selected cold-pressed oils’ addition on the thermal degradation of lipophilic bioactive compounds in the obtained oil blends and their antiradical properties determined by the 2,2 diphenyl-1-picrylhydrazyl (DPPH) and 2,2′-azino-bis(3-ethylbenzothiazoline-6-sulphonic acid) (ABTS) methods were investigated.

## 2. Materials and Methods

### 2.1. Research Material

Refined rapeseed oil (Bunge Polska Sp. z o. o., Kruszwica, Poland) was purchased at a local grocery store. Cold-pressed rapeseed oils (Olini, Miramar Sp. z o.o., Wałbrzych, Poland), cold-pressed coriander seed oil (Efavit, Poznań, Poland) and cold-pressed apricot kernel oil (Olini, Miramar Sp. z o.o., Wałbrzych, Poland) were purchased directly from the local cold-pressing factories.

### 2.2. Blends Preparation

Refined rapeseed oil without additives (RefO) and refined rapeseed oil with the addition of 200 mg TBHQ/kg oil (rTBHQ) were the negative and positive controls, respectively. Six types of blends were prepared by blending refined rapeseed oil with cold-pressed rapeseed oil (RO5% and RO25%), cold-pressed coriander seed oil (CO5% and CO25%) and cold-pressed apricot kernel oil (AO5% and AO25%), each at a concentration of 5 and 25% (*v/v*).

The free space in the bottle was filled with nitrogen. After, the samples were mixed for 1 h at room temperature using an IKA, KS 501 shaker (IKA, Staufen, Germany). Next, samples were left for 24 h to distribute the additive evenly. The samples were prepared in dark, glass bottles. The blends were stored in refrigerated conditions (4 °C) until the next stages of research.

### 2.3. Heating Procedure

Each sample of blends or refined rapeseed oil was heated at 170 and 200 ± 10 °C. Briefly, 50 mL of each oil was heated in a thin layer on a pan with a diameter of 20 cm using electrical hotplates MS-H-Pro (Scilogex, Rocky Hill, CT, USA) with an external temperature control. Each heating process was performed in two repetitions. At first, oils were preheated for 7 min to achieve 170 °C or for 9 min to achieve 200 °C. Next, the heating of the oils process was continued for 10 min, maintaining a temperature of 170 or 200 °C. Time and temperature applied were typically for the frying process, according to previous studies [49,50,51,52]. The actual oil temperature was verified constantly, using an electronic Testo mini surface thermometer (Testo SE & Co., KGaA, Titisee-Neustadt, Germany) in three points on the pan. After chilling, the oil samples were transferred to plastic containers in which the remaining free space was filled with nitrogen. Closed sample containers were stored under nitrogen, without access to light and at a temperature of −30 °C until the analysis.

### 2.4. Fatty Acid Profile

The composition of fatty acids was analyzed according to the AOCS Ce 1h-05 method [53]. Oil samples were dissolved in hexane and transesterified to fatty acid methyl esters (FAME) using sodium methoxide solution in methanol. The assay was carried out by an Agilent 7820A GC gas chromatograph (Agilent Technologies, Santa Clara, CA, USA) equipped with a flame ionization detector (FID) and an SLB-IL111 capillary column (Supelco, Bellefonte, PA, USA) (100 m; 0.25 mm; 0.20 μm). The Fatty Acid Methyl Ester (FAME) Mix standard (Supelco, Bellefonte, PA, USA) was applied for the identification of FAME peaks. The results were expressed as the percentage of individual acids in the oil sample.

### 2.5. Phytosterols Analysis

The analysis of phytosterols was performed following the previously described methodology [54]. The oil samples with the internal standard (5a-cholestane) were saponified with 1 M KOH in methanol at room temperature for 18 h. The unsaponifiable fraction was extracted twice with hexane/methyl tert-butyl ether (1:1, *v/v*). The methyl tert-butyl ether layer was transferred to a separate test tube and the solvent was evaporated under nitrogen. Silylating reagent (BSTFA with 1% TCMS, Thermo Scientific, Waltham, MA USA) and pyridine (Sigma-Aldrich, St. Louis, MO, USA) were added to the obtained samples and analyzed using an Agilent 7820A GC chromatograph (Agilent Technologies, Wilmington, DE, USA) equipped with a flame ionization detector (FID) and DB-35MS capillary column (Agilent Technologies, 30 m, 0.20 mm, 0.33 μm). The analytical conditions were as follows: the initial oven temperature was 100 °C for 5 min, then increased 25 °C/min to 250 °C and after 3 °C/min to 290 °C, the temperature of the detector (FID) was 250 °C; the carrier gas was helium at a flow of 1 mL/min.

### 2.6. Tocochromanols Analysis

**Figure 1:**
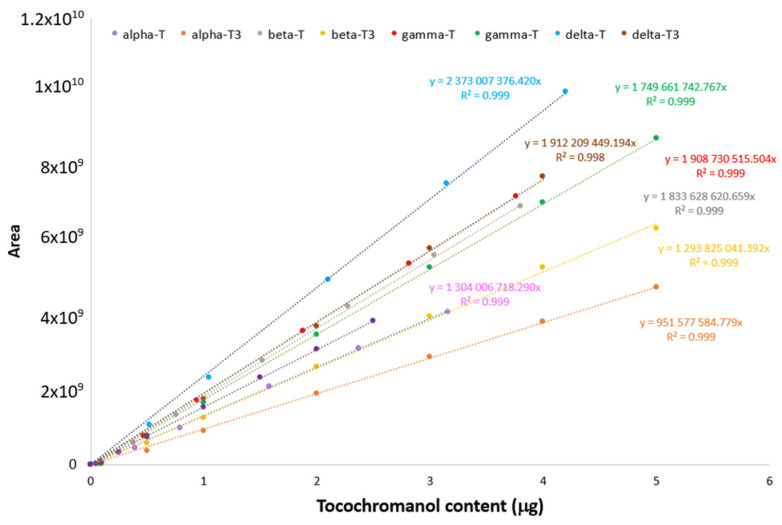
Calibration curves for quantification of tocochromanols.

The composition of tocochromanols and their total content were determined according to Siger et al. [55]. The oil was dissolved in n-hexane and transferred to vials. The tocochromanol content was analyzed using the Waters HPLC system (Waters, Milford, MA, USA) equipped with a LiChrosorb Si 60 column (Merck, Darmstadt, Germany) (250 mm, 4.6 mm, 5 µm) and a fluorimetric detector (Waters 474). Additionally, a photodiode array detector (Waters 2998 PDA) was used for qualitative determination. The mobile phase was a mixture of n-hexane with 1.4-dioxane (96:4 *v/v*). The flow rate was 1.0 mL/min (for tocochromanols). To detect the fluorescence of tocochromanols, the excitation wavelength was set at λ = 295 nm and the emission wavelength at λ = 330 nm. Each compound was quantified using the calibration curve for the corresponding standard (Figure 1). Standards of all tocochromanols (>95% of purity) were purchased from Merck (Darmstadt, Germany).

### 2.7. Chlorophyll Pigments Analysis

Chlorophyll pigments were analyzed using the AOCS Cc 13i-96 method [56]. Briefly, the determination was performed directly in the oil sample at 670 nm in a 10 mm spectrophotometer cell against air. Results were expressed as mg of pheophytin in 1 kg of oil.

### 2.8. Determination of Radical Scavenging Activity

The extraction procedure for the preparation of methanolic extracts of the studied oils and their radical scavenging activity (RSA) was determined according to two analytical methods, DPPH and ABTS, described in detail by Szydłowska-Czerniak et al. [57].

#### 2.8.1. Extraction Procedure

The test tubes with oils (3.00 g) and methanol (5 mL) were shaken for 30 min using a shaker SK-0330-PRO (CHEMLAND, Stargard, Poland) at room temperature. The extracts were separated from oils in a freezer (−20 °C, 30 min) and transferred quantitatively into glass bottles. Each oil sample was extracted in triplicate and extracts were stored in a refrigerator until the analysis.

#### 2.8.2. DPPH Method

Briefly, 0.25–0.50 mL of oil extracts was added to 1.75–1.50 mL of methanol, respectively, and 0.5 mL of DPPH methanolic solution (304.0 μmol L^−1^). The mixtures were shaken vigorously and left in the dark for 15 min. The absorbance was measured at λ = 517 nm against a reagent blank (2 mL of methanol + 0.5 mL of DPPH methanolic solution) using a Helios a spectrophotometer (Unicam, Cambridge, UK) in a 1 cm quartz cell.

#### 2.8.3. ABTS Method

The cation radical ABTS^•+^ solution was prepared by mixing 7 mmol L^−1^ ABTS solution and 2.45 mmol L^−1^ K_2_S_2_O_8_ in a 2:1 ratio and kept for 16 h. Next, ABTS^•+^ solution was diluted with ethanol to an absorbance of 0.70 at 734 nm; 0.05–0.10 mL of oil extracts was added to 2.45–2.40 mL of ABTS^•+^ solution and the mixtures were incubated at 30 °C and in darkness for 5 min. The absorbance was measured at 734 nm against a reagent blank (2.5 mL of ABTS^•+^ solution).

### 2.9. Statistic Calculation

The heating process of the blends was carried out on two independent pans. All analyses were performed in duplicate. Results of fatty acids composition, phytosterols, tocochromanols and chlorophyll for unheated blends and cold-pressed oils were presented as the average of two determinations ± standard deviation (SD) and for heated blends as the average of four determinations ± standard deviation (SD). The RSA results of the unheated blends and cold-pressed oils were expressed as an average of three determinations ± standard deviation (SD), while RSA values for the heated blends were the average of six repetitions ± SD. The statistical significance of differences between means was determined using one-way analysis of variance (ANOVA) and the Tukey test (*p* < 0.05). Pearson’s correlation analysis was applied to calculate the relationships between RSA and amounts of bioactive compounds. Multivariate statistical analysis was performed using principal component analysis (PCA). Calculations and charts were made in Statistica 13.3 and MS Excel 2019.

## 3. Results and Discussion

### 3.1. Fatty Acids

**Figure 2:**
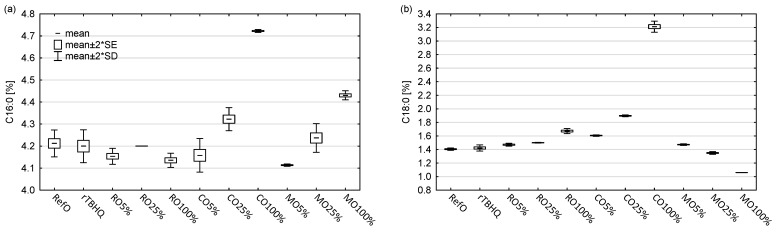

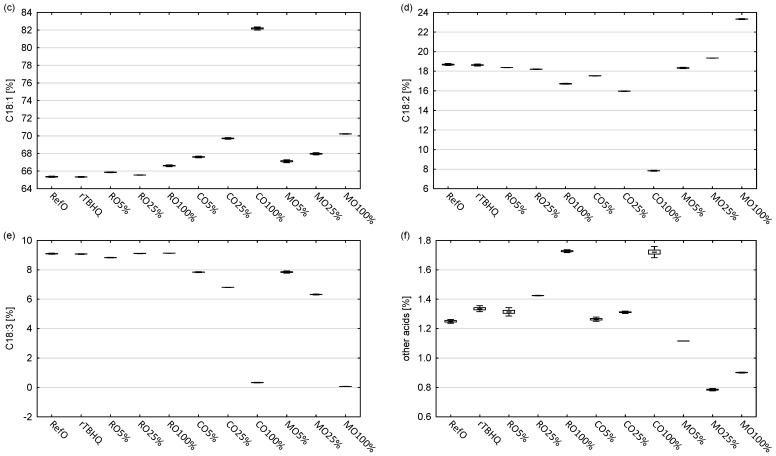
Main fatty acids share in non-heated blends and cold-pressed oils: (**a**) palmitic acid (C16:0); (**b**) stearic acid (C18:0); (**c**) oleic and petroselinic acids sum (C18:1); (**d**) linoleic acid (C18:2); (**e**) α-linolenic acid (C18:3); (**f**) sum of C14:0, C16:1, C20:0, C20:1, C22:0, C22:1 (other acids).

The fatty acid profile is presented in Figure 2. The predominant share had oleic acid (C18:1 n-9). It constituted 65.36, 65.34, 66.61 and 70.22% in control samples RefO and rTBHQ and in cold-pressed oils RO100% and AO100% respectively. The oleic acid share ranged from 65.56 (RO25%) to 67.97% (AO25%). Based on previous studies [58,59], it may be assumed that over 70% of fatty acid in coriander seed oil (CO100%) constituted rare petroselinic acid (C18:1 n-12). It was found that C18:1 (oleic and petroselinic acids sum) shared 82.18, 69.71 and 67.60% in CO100%, CO25% and CO5%, respectively. The next fatty acid was linoleic (C18:2; 15.96–19.34%), followed by α-linolenic (C18:3; 6.32–9.11%), palmitic (C16:0; 4.11–4.32%) and stearic (C18:0; 1.35–1.90%). The total share of other identified acids did not exceed 2%. The fatty acid profile of cold-pressed oils and refined rapeseed oil was typical for their species [32,60]. The addition of cold-pressed oils caused changes in the acid profiles of the blends compared to refined rapeseed oil, and they were most visible in the blends with coriander seed oil and apricot kernel oil. The addition of cold-pressed oils caused an increase in the C18:1 share in CO5%, CO25%, AO5% and AO25% by 2.24%, 4.35%, 1.76% and 2.61%, respectively. The addition of coriander seed oil resulted in a decrease in the C18:2 share by 1.15% (CO5%) and 2.72% (CO25%) as well as a decrease in the C18:3 share by 1.26% (CO5%) and 2.30% (CO25%). In blends with apricot kernel oil, the share of C18:3 decreased by 1.25% in AO5% and by 2.78% in AO25%. The changes of fatty acids during heating was presented at Supplementary Materials (Table S1).

### 3.2. Phytosterols

The highest phytosterol content was observed in cold-pressed rapeseed oil RO100% (500.97 mg/100 g oil) (Table 1). In the non-heated blends and control samples, phytosterol content ranged between 425.65 (AO25%) and 498.05 mg/100 g oil (RO5%). In all the heated blends and control samples, the most prevalent phytosterols were β-sitosterol (232.69–248.84 mg/100 g oil) and campesterol (136.50–175.14 mg/100 g oil), which together accounted for 81.85–86.74% of the total phytosterols. Brassicasterol was third in terms of content (43.61–72.85 mg/100 g oil). It was found in blends, control samples and cold-pressed rapeseed oil, but it was not identified in the cold-pressed coriander seed and apricot kernel oils. ∆^5^-avenasterol was found in all samples with the highest content in cold-pressed apricot kernel oil AO100% (22.40 mg/100 g oil), AO25% (8.96 mg/100 g oil) and CO100% (6.10 mg/100 g oil). CO100% and its blends contained rare phytosterols Δ^7^-stigmasterol (30.73 mg/100 g oil), Δ^7^-avenasterol (15.73 mg/100 g oil) and citrostadienol (15.25 mg/100 g oil). ∆^7^-avenasterol was also detected in CO25% (5.69 mg/100 g oil) and AO100% (3.63 mg/100 g oil).

The phytosterol content in coriander seed oil and apricot kernel oils in the current study [61,62,63] was higher than in oils presented in some papers. However, cold-pressed rapeseed and apricot kernel oils were less abundant in phytosterols, compared to the next article [32]. According to Ramadan et al. [63], the most dominant phytosterol cold-pressed coriander seed oil was β-sitosterol (1.182 g/kg), next stigmasterol (1.512 g/kg) and ∆^5^-avenasterol (1.466 g/kg). The content of other individual phytosterols did not exceed 0.750 g/kg. In the studies on apricot kernel oil [61] and rapeseed oil [32], the most prevalent phytosterol was β-sitosterol containing 1555 μg/g (over 88% total phytosterols) and 3656.50 mg/kg (over 45% total phytosterols), respectively. Next was campesterol containing 99 μg/g (over 5% total phytosterols) and 2979.80 mg/kg (over 37% total phytosterols), respectively.

**Figure 3:**
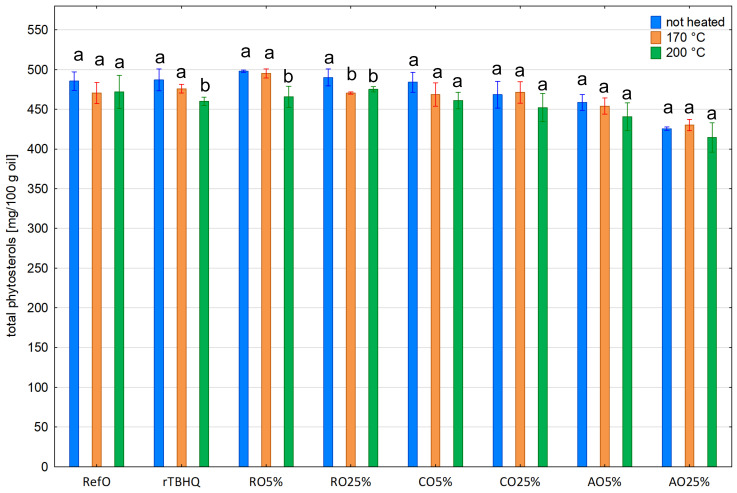
Total phytosterol content in non-heated and heated blends. Different letters indicate significant differences (*p* < 0.05) between samples not heated and heated at 170 and 200 °C.

Overall, heating at 170 and 200 °C did not result in a statistically significant reduction in the phytosterol content (Figure 3). Only in samples RO25% heated at 170 °C and rTBHQ, RO5% and RO25% heated at 200 °C exposed a statistically significant decrease in the phytosterol content (96.00; 94.46; 93.54; 96.97% respectively). Also, considering the individual content of the two main phytosterols, β-sitosterol and campesterol, no statistically significant changes were observed. The exception was the samples rTBHQ heated at 200 °C, in which the contents of β-sitosterol and campesterol statistically significantly differed compared to the unheated sample. Δ^7^-stigmasterol turned out to be the least stable phytosterol both during heating at 170 °C and 200 °C.

Generally, the obtained data were consistent with the previous study on the thermal stability of sterols in vegetable oils and margarines [64]. They concluded that the total loss of phytosterols after heating at 150–210 °C for 8–16 min was too small to directly quantify. Another article [65] reported 2–6% phytosterol loss of the original amount in liquid margarine enriched with phytosterol esters after pan frying at 160–200 °C for 5–10 min. However, Chen et al. [66] observed significant phytosterol loss during heating of different lipid matrixes at a temperature of 120–180 °C and time of 30–180 min. Moreover, Salta et al. [67] noted a considerable loss of total and individual phytosterols during repeated deep and pan frying. These discrepancies are probably due to differences in process conditions such as time, temperature, surface to volume ratio and frying food. It should be noted that samples with the highest and significant phytosterol content decreasing during heating had the highest phytosterol initial content and initial activity DPPH. It may be speculated above some necessary concentration of phytosterols that the exhibited protection activity against TAG is by sacrificing and degrading themselves. It was shown that the phytosterol loss is caused by phytosterol oxidation [68]. However, the protection activity should have another mechanism than that of being an antioxidant, because correlation and PCA analysis did not show a relationship between phytosterol content and RSA analyzed by DPPH assay. Another possible, partial explanation, though less often considered, is phenol acid triterpene alcohol and phytosterol conversion to oryzanol ester under high temperatures [69].

### 3.3. Tocochromanols

The highest content of tocochromanols was found in RefO (70.45 mg/100 g oil) and rTBHQ (69.24 mg/100 g oil) (Table 1). The dominant homologs were γ-tocopherol, with the highest content in AO100% (54.30 mg/100 g oil) and AO25% (36.72 mg/100 g oil), and α-tocopherol with the highest content in CO100% samples (50.68 mg/100 g oil) and CO25% (36.66 mg/100 g oil). AO100% and CO100% were characterized by the highest content of δ-tocopherol (1.65 mg/100 g oil) and β-tocopherol (1.39 mg/100 g oil), respectively. The presence of tocotrienols was noticed in CO100% and its blends. Their main tocotrienol homolog was γ-tocotrienol containing 3.07, 0.72 and 0.15 mg/100 g of oil, respectively. The results of the tocochromanol content and composition in cold-pressed apricot kernel oil were consistent with other authors’ studies. According to Hassanien [61] and Ying [62], total tocochromanol content in apricot kernel oil was 589.0 μg/g and 59.9 mg/100 g, respectively. γ-Tocopherol was the most prevalent homolog in both articles. The next paper on coriander seed oil [33] noted lower total tocochromanols content (327.47 μg/g) and different dominant tocochromanol homolog (γ-tocotrienol, 238.40 μg/g) compared to this investigation.

**Figure 4:**
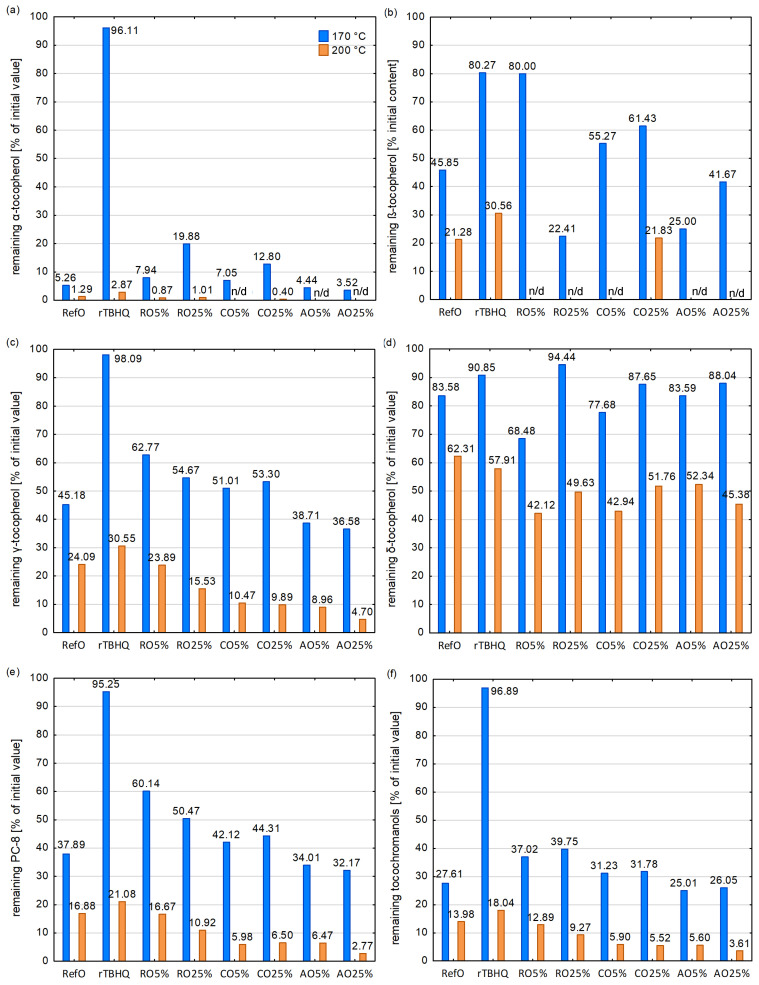
The stability of tocochromanols and individual homologues in samples heated at 170 and 200 °C: (**a**) α-tocopherol; (**b**) β-tocopherol; (**c**) γ-tocopherol; (**d**) δ-tocopherol; (**e**) plastochromanol-8; (**f**) total tocochromanols; n/d—not detected.

**Figure 5:**
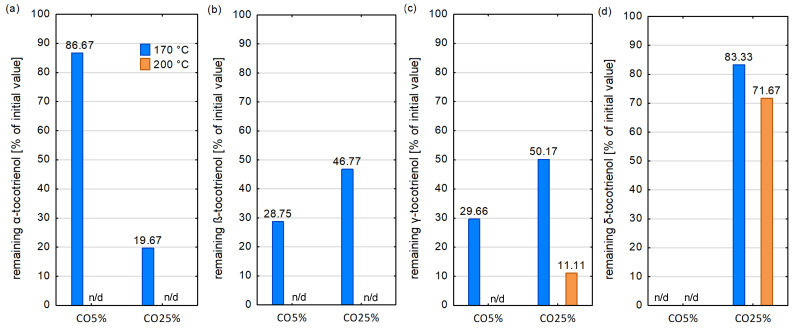
The stability of tocotrienols in CO5% and CO25% heated at 170 and 200 °C; n/d—not detected: (**a**) α-tocotrienol; (**b**) β-tocotrienols; (**c**) γ-tocotrienol; (**d**) δ-tocotrienol.

At temperatures of 170 °C (Figure 4 and Figure 5), rTBHQ was characterized by the highest amount of tocochromanols and individual homologues stability. Among other samples, the highest remaining amount of tocochromanols, γ-tocopherol and PC-8 were reported in RO5% samples (37.02, 62.77 and 60.14% of the initial content, respectively) and in RO25% (39.75, 54.67 and 50.47% of the initial content, respectively). Moreover, β- and δ-tocopherol were more stable in the RO5% sample than in the RefO. However, blends with coriander seed and apricot kernel cold-pressed oils showed a lower or comparable stability of tocochromanols than RefO.

rTBHQ, RefO and RO5% exhibited the highest remaining amount of tocochromanols at 200 °C (18.04, 13.98 and 12.89% of the initial tocochromanol content, respectively). In the other samples, the final content of tocochromanols after heating dropped below 10% of the initial content. rTBHQ, RefO and RO5% samples had the highest stability of γ-tocopherol and PC-8. In CO5%, AOR5% and AO25% heated at 200 °C α- and β-tocopherol were completely decomposed. The changes of tocochromanols content during heating process was presented at Supplementary Materials (Table S2).

In the current investigation, individual homologue tocopherol stability was according to the following order: δ > γ > β > α, which was consistent with other research during deep and pan frying [70] and at storage conditions [42]. Cui et al. [71] observed that the content of α-tocopherol decreased while the γ-tocopherol and δ-tocopherol content remained stable or even increased slightly, respectively. When the α-tocopherol was completely lost, δ-tocopherol and γ-tocopherol began to be reduced. It leads to the conclusion that at the first stage of the thermal treatment process, mainly α-tocopherol plays a protective role against TAG. In the next stage, when α-tocopherol is degraded, γ-tocopherol and δ-tocopherol take over the protection function. Athanasiadis et al. [42] stated that the synergism between the tocopherols protects them from the oxidation process. Considering tocopherol stability in the lipid matrix, the tocopherol mixture is more beneficial than individual homologues occurring. Winkler-Moser et al. [72] remarked that the α-tocopherol initial content decreased with a lower rate in the presence of α-tocotrienol, which resulted in a significantly higher final α-tocopherol retention. However, it should be noted that some researchers have obtained findings different to ours. Rodríguez et al. [73] showed that the order of the degradation rates was shaped as follows: γ > δ > α. Xu et al. [74] reported that γ-tocopherol was less stable than α-tocopherol during thermal treatment of high oleic oils and palm oil. It could be explained that tocochromanol stability depends not only on their structure, but also on their content and homologues with a higher content would degrade much faster than those with a lower content [74]. Generally, it is common knowledge that tocochromanols, especially α-tocopherol, may have a prooxidant effect in vegetable oils if they have concentrations that are too high [75,76]. Another possible explanation of this phenomenon may be that for each homologue, there is some optimal concertation, above or below, which its stability decreased. Moreover, many factors, like time, temperature process and pan- or deep-frying conditions may influence the reaction mechanism occurring in oils during thermal treatment.

### 3.4. Chlorophyll

The highest chlorophyll content was found in samples CO100% (1.32 mg/100 g oil) and RO100% (1.07 mg/100 g oil) (Table 1). In coriander and rapeseed blends, chlorophyll content ranged from 0.04 to 0.33 mg/100 g oil. In another study [77], rapeseed oil chlorophyll content varied between 0.9 and 51.0 mg/kg. According to the authors, these differences could result from the climatic variations in different parts of the region and differences in the maturation stage at the time of harvest. They stated that immature seeds contain high amount of chlorophyll. However progressive metabolism of chlorophyll during seeds ripening leads to a reduction in chlorophyll final level. It was shown in [78] that the chlorophyll content fell from 1239 mg/kg at the beginning, to 4 mg/kg in the fully matured seeds (after 35 days). The next experiment [79] researched rapeseed oils fortified with 1 and 5% sesame, chia, hemp seeds and hemp herb. The samples were stored in the dark and light for 3 months. The chlorophyll content was 0.17 mg/kg at the beginning. After 3 months, it ranged between 0.14 (control sample and sample fortified with 1% of chia seeds) to 1.20 mg/kg (sample fortified with hemp herb) in samples stored in the dark, while it was a complete loss in samples stored in light. There are little data on the chlorophyll content of coriander seed oil. So far, available publications reported higher levels at 11.1 mg/kg [58] or 23.43 mg/kg [60] of AO25%.

### 3.5. DPPH and ABTS Radical Scavenging Activity and Their Correlation with Bioactive, Lipophilic Compounds

DPPH and ABTS results of non-heated control samples and oil blends ranged between 172.89 and 313.78 μmol TE/100 g and 804.77 and 984.56 μmol TE/100 g, respectively (Table 1). However, DPPH and ABTS values for cold-pressed oils varied from 332.35 to 435.41 μmol TE/100 g and 796.84–1268.65 μmol TE/100 g, respectively. Both DPPH and ABTS methods are based on the reduction of stable purple radical DPPH and blue–green radical cation ABTS in the presence of hydrogen-donating antioxidants due to the formation of the colourless non-radical forms DPPH-H and ABTS-H, respectively, in the reactions. DPPH and ABTS assays are mixed-mode tests using both hydrogen atom transfer (HAT) or single-electron transfer (SET) mechanisms, depending on the corresponding reaction conditions. ABTS assay is used to determine scavenging ability both in hydrophilic and lipophilic antioxidant systems, whereas DPPH assay applies a radical dissolved only in organic media, and therefore, it suits lipophilic systems [80]. It is probably the reason that CO100% and AO100% had lower DPPH value compared to rTBHQ (42.18 and 63.03%, respectively) (Table 1). However, ABTS results were comparable (no significant difference between CO100% and rTBHQ) or showed less difference (decrease by 18.32% comparing AO100% to rTBHQ). Among the non-heated samples, RO100% had the lowest ABTS value. On the other hand, the RO100% ability to DPPH radical scavenging was relatively high compared to other non-heated samples, and a higher DPPH value was found only for rTBHQ and CO100%. It may be assumed that CO100% and AO100% were better ABTS scavengers than RO100%; thus, other compounds with hydrophilic properties, not determined in this study, that were present in their mixtures, affected their ability for ABTS scavenging. The obtained DPPH and ABTS results showed that antioxidants in RO100% had mainly hydrophobic properties. However, it is noteworthy that natural antioxidants present in RO100% were weaker DPPH scavengers than synthetic TBHQ added to refined rapeseed oil (DPPH = 332.35 and 752.99 μmol TE/100 g, respectively). Moreover, the DPPH scavenging activity of RefO was significantly lower compared to RO100% (by 97.35%), CO100% (by 158.54%) and AO100% (by 65.30%). As expected, all investigated oil blends revealed a significantly higher ability to scavenge DPPH and ABTS radicals than RefO (by 14.42–86.32% and 9.57–21.11%, respectively), except DPPH for AO5% and ABTS for RO5%, RO25%.

**Figure 6:**
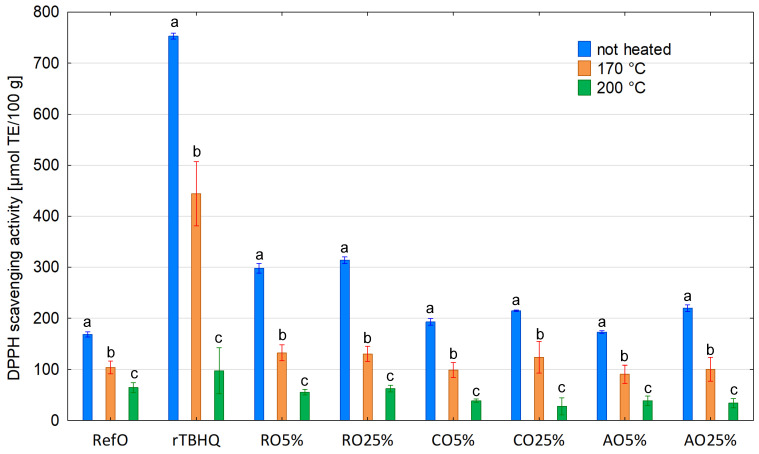
DPPH scavenging activity of non-heated and heated blends. Different letters indicate significant differences (*p* < 0.05) between samples not heated and heated at 170 and 200 °C.

**Figure 7:**
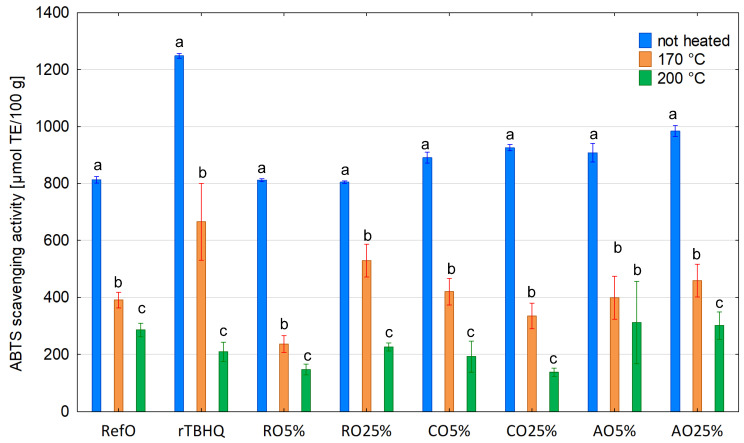
ABTS scavenging activity of non-heated and heated blends. Different letters indicate significant differences (*p* < 0.05) between samples not heated and heated at 170 and 200 °C.

Heating at both the studied temperatures caused a significant decrease in DPPH and ABTS results for all oil samples (Figure 6 and Figure 7). At 170 °C, DPPH and ABTS values ranged between 90.75 and 132.28 μmol TE/100 g as well as 236.51 to 528.70 μmol TE/100 g, respectively. Among the samples heated at 170 °C, the highest RSA stability determined by DPPH and ABTS was observed for RefO (59.02% of initial value) and RO25% (65.70% of initial value), respectively, while the lowest stability of DPPH and ABTS radicals’ scavenging was found for RO25% (41.51% of initial value) and RO5% (29.13% of initial value), respectively. The treatment of oil samples at a higher temperature (200 °C) decreased DPPH (27.94–62.54 μmol TE/100 g) and ABTS values (137.78–311.55 μmol TE/100 g) more intensely. At 200 °C, the stability of DPPH radical scavenging ability amounted to 23.67, 19.95 and 10.39% of the initial value for RefO, AO5% and CO5%, respectively. For the remaining samples, the stability to scavenge the DPPH radical was below 10% of the initial value, whereas at 200 °C, the stability to scavenge the ABTS radical cation ranged between 5.38 and 18.45% and decreased to below 10% for rTBHQ, RO5% and CO25%.

It is clear that the degradation of tocochromanols resulted in a decrease in antiradical activity under heating. It is consistent with previous results obtained by other authors [81,82,83,84]. Generally, the stability of DPPH and ABTS scavenging activity corresponded to the stability of tocochromanols. This is confirmed by the results of the correlation analysis (Table 2), which showed a strong positive correlation between DPPH value and total tocochromanols (0.7552), and a very strong positive correlation between ABTS value and total tocochromanols (0.9093). However, the former was lower than expected. Moreover, surprisingly, the former was lower compared to the latter. The higher correlation tocochromanol content with ABTS results could be explained by some synergy interaction between hydrophilic and hydrophobic antioxidants [85]. The other reason may be generated during heating new compounds showing antioxidant activity [86]. Furthermore, the DPPH radical could react with some products of thermal degradation of oil [87], which could cause some unexpected artefacts. Among tocopherol homologues, α-tocopherol showed the strongest, positive correlation with RSA determined by DPPH and ABTS methods (r = 0.7245 and r = 0.8476 respectively). On the other hand, the remaining tocopherol homologues showed only a moderate or weak correlation with DPPH and ABTS values. These results confirm previous studies [88], which showed that α-tocopherol is the predominant chain-breaking antioxidant, and it may be explained by the lowest bond dissociation energy of the phenolic hydrogen in α-tocopherol. Noteworthy, the correlation between RSA and α-tocopherol content was lower compared to the correlation between RSA and total tocohromanols. It may be contributed by a synergy between tocopherol homologues, which together exhibit more antioxidant properties than individual homologues. Therefore, a tocopherol mixture is more efficient in the retardation of oil degradation than only one or two homologues. Moreover, no correlation or a weak, negative correlation was found between DPPH or ABTS values and total phytosterols (r = −0.1273 and −0.3488, respectively). On this account, phytosterols showed no antioxidant properties in the condition of current experiments.

In the work of other authors [89], it was noted that tocopherols—phytosterols and tocopherols—γ-oryzanol mixtures added to rice bran oil showed antagonistic effects on the results of a Rancimat test. However, the same authors in another publication [90] found that the same mixtures exhibited synergistic interactions in refined coconut oil. The authors conclude that the same combination of two bioactive substances, depending on the lipid environment in which it occurs, may cause different interactions and thus may show different antioxidant properties. The authors also noted that the type of interaction depended on the proportions between the mixture ingredients as well as the concentration of the mixtures in oils. These conclusions are consistent with our results on the correlation between antioxidant activity and content of bioactive substances. Moreover, the authors stated that further research is required to better understand the observed phenomena. Ramírez-Anaya et al. [91] compared the antioxidant capacity of extra virgin olive oil used to treat potatoes, eggplant, tomato and pumpkin by different techniques. They observed a significant decrease in DPPH and ABTS radical scavenging activities after the sautéing technique and DPPH radical scavenging activities after deep frying. Surprisingly, an increase in ABTS radical scavenging activities was found after deep frying potato, pumpkin and tomato, but the authors did not explain that phenomenon. Interestingly, a previous study on a synthetic phenolipids’ effect on rapeseed oil stability during frying [83] observed an increase in DPPH and ABTS results. It was attributed to the migration of antioxidant substances from fried food to frying medium. Additionally, octyl esters under the influence of high temperatures could be degraded and hydrolyzed to phenolic acids with high antioxidant activity. Also, it should be highlighted that the composition of bioactive substances as well as DPPH and ABTS results varies in different works and may depend on many factors, such as agrotechnical and weather conditions during cultivation, variety, maturity of seeds and the method of their storage, as well as the way and parameters of the oil production process.

### 3.6. Principal Component Analysis (PCA)

The PCA model retained three principal components (PC1, PC2 and PC3), which gave eigenvalues greater than 1.00 (5.79, 4.95 and 2.67, respectively). They described 89.17% of the total variability (38.61%, 33.02% and 17.77%, respectively).

PC1 was mainly characterized by content of β-tocopherol (−0.9657) and C18:1 share (−0.9167) (Table 3). Otherwise, PC2 was highly correlated with tocochromanols (0.9350), and PC-8 content (0.9037). ABTS and DPPH values and α-tocopherol content showed a strong correlation with PC2 (0.7995, 0.7376 and 0.7125, respectively). The strongest, negative correlation with the PC3 was noted for the sum of other acids (−0.7154) and C18:3 share (−0.6910) and phytosterol content (−0.6838).

**Figure 8:**
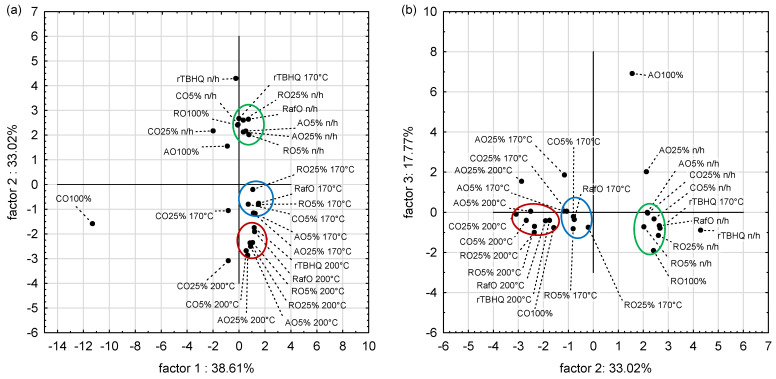
Principal component analysis score plot describing the relationship between non-heated (n/h,) and heated at 170 and at 200 °C samples (**a**) PC1 and PC2, (**b**) PC2 and PC3.

The results of sample grouping were presented in the score plots (Figure 8). The score plot on PC1 and PC2 showed that blends were distributed along the y-axis (assigned to the PC2), from blends heated at 200 °C, next the blends heated at 170 °C, and finally the unheated blends. The share of C18:1 was placed on the left side of the PC1, so samples located on this side showed a higher C18:1 share. The score plot on PC2 and PC3 revealed that a rising value of PC3 score was associated with a lowering C18:3 share in samples. Three separate clusters were distinguished in the score plot on PC1 and PC2. The first cluster contained most of the unheated samples and additionally, rTBHQ heated at 170 °C (green). The second (blue) and third (red) clusters included samples heated at 170 °C and 200 °C, respectively. Noteworthy, clusters of samples heated at 170 °C and 200 °C were separated from each other, but located in the same quarter, and far from the cluster of unheated samples, which was placed in another quarter. Results of PCA analysis indicated that heating caused significant changes in the samples, and it was more expanded at 200 °C. However, the diversity of samples unheated and heated at 170 °C was greater than in samples heated at 170 and 200 °C. The arrangement of samples at the PC2 and PC3 score plot was likewise the PC1 and PC2 score plot, which confirms the results of previous analysis of the PC1 and PC2 score plot. Moreover, it should be noted that oil CO100% and blend CO25% on the PC1 and PC2 score plot as well as AO100% and AO25% on the PC2 and PC3 score plot were separated from the others and it was associated with differences in fatty acid composition.

## 4. Conclusions

The heating caused a significant decrease in tocochromanol content and a reduction in RSA at both temperatures wherein it was more substantial at 200 °C. Correlations between the DPPH or ABTS values and total tocochromanols as well as α-tocopherol were observed. However, there were weak or modest correlations between RSA determined by DPPH or ABTS methods and remaining tocopherol homologues. Our results revealed synergy interactions between individual tocopherol homologues, which in combination showed higher correlations with RSA than individual homologues. Moreover, the synergy interaction between hydrophobic and hydrophilic antioxidants may be assumed to occur. Generally, heating had only a minor effect on the decrease in phytosterol content. No correlation between the DPPH or ABTS values and total phytosterols was found. However, the blends with the highest initial phytosterol content showed that the highest phytosterol losses during heating and phytosterols could exhibit a protecting activity, but based on mechanisms other than antioxidant activity. This theory should be confirmed in further studies. The obtained results may be useful in designing a new type of frying oil for food with improved health properties and it may be the basis for further research on this topic.

## Figures and Tables

**Table 1:** Characteristic parameters of non-heated oils and blends of cold-pressed oils.

	RefO	rTBHQ	RO5%	RO25%	RO100%	CO5%	CO25%	CO100%	AO5%	AO25%	AO100%
phytosterols [mg/100 g oil]											
brassicasterol	62.10 ± 1.44^c^	62.94 ± 0.56^c^	63.05 ± 0.10^c^	62.33 ± 5.88^c^	57.63 ± 2.57^bc^	49.99 ± 0.77^ab^	n/d	54.42 ± 2.46^bc^	43.61 ± 1.43^a^	n/d	62.10 ± 1.44^c^
campesterol	165.07 ± 5.19^d^	171.08 ± 4.14^d^	175.14 ± 0.34^d^	170.81 ± 2.44^d^	171.73 ± 3.10^d^	168.11 ± 7.97^d^	149.21 ± 5.78^bc^	26.68 ± 0.48^a^	161.81 ± 3.07^cd^	136.50 ± 0.25^b^	14.26 ± 1.46^a^
campestanol	3.91 ± 0.51^ab^	4.76 ± 0.22^b^	4.94 ± 0.38^b^	5.46 ± 0.45^b^	5.23 ± 1.18^b^	4.40 ± 0.23^ab^	4.52 ± 0.38^ab^	5.57 ± 0.38^b^	4.04 ± 0.42^ab^	3.89 ± 0.81^ab^	2.25 ± 0.84^a^
stigmasterol	n/d	n/d	n/d	n/d	n/d	3.32 ± 0.26^a^	7.94 ± 0.39^b^	25.93 ± 0.11^c^	n/d	n/d	n/d
ß-sitosterol	238.88 ± 4.79^c^	244.02 ± 6.99^c^	248.84 ± 1.40^c^	244.34 ± 8.01^c^	251.13 ± 5.35^c^	241.38 ± 7.61^c^	234.15 ± 8.67^c^	129.2 ± 0.31^a^	233.32 ± 4.07^c^	232.69 ± 0.99^c^	200.67 ± 1.67^b^
Δ^5^-avenasterol	4.98 ± 0.07^a^	5.17 ± 0.76^a^	6.20 ± 0.48^a^	6.40 ± 0.46^ab^	10.55 ± 3.20^b^	5.28 ± 0.81^a^	6.92 ± 0.26^ab^	6.10 ± 0.25^a^	5.14 ± 0.05^a^	8.96 ± 0.48^ab^	22.4 ± 0.02^c^
Δ^7^-stigmasterol	n/d	n/d	n/d	n/d	n/d	3.99 ± 0.26^b^	5.71 ± 0.65^c^	30.73 ± 0.25^d^	n/d	n/d	2.76 ± 0.02^a^
Δ^7^-avenasterol	n/d	n/d	n/d	n/d	n/d	n/d	5.69 ± 0.08^b^	15.73 ± 1.25^c^	n/d	n/d	3.63 ± 0.77^a^
citrostadienol	n/d	n/d	n/d	n/d	n/d	n/d	4.24 ± 0.09^a^	15.25 ± 0.01^b^	n/d	n/d	n/d
total phytosterols	485.68 ± 11.61^cd^	487.13 ± 13.54^cd^	498.05 ± 1.28^d^	490.06 ± 10.56^cd^	500.97 ± 5.66^d^	484.11 ± 12.50^cd^	468.38 ± 16.9^cd^	255.2 ± 1.45^a^	458.74 ± 9.98^bc^	425.65 ± 1.97^b^	245.97 ± 1.39^a^
tocochromanols [mg/100 g oil]											
α-tocopherol	30.70 ± 0.05^e^	30.18 ± 0.48^e^	25.2 ± 0.23^c^	28.18 ± 0.11^d^	26.41 ± 0.24^c^	29.6 ± 0.45^e^	36.66 ± 0.48^f^	50.68 ± 0.49^g^	25.45 ± 0.12^c^	20.76 ± 0.28^b^	1.66 ± 0.00^a^
β-tocopherol	0.13 ± 0.03^ab^	0.16 ± 0.02^b^	0.05 ± 0.01^ab^	0.15 ± 0.05^ab^	0.09 ± 0.01^ab^	0.19 ± 0.01^b^	0.43 ± 0.05^c^	1.39 ± 0.08^d^	0.11 ± 0.03^ab^	0.06 ± 0.01^ab^	n/d
γ-tocopherol	33.58 ± 0.09^e^	32.78 ± 0.95^de^	24.15 ± 0.16^b^	31.78 ± 0.26^cd^	32.84 ± 0.25^de^	31.23 ± 0.22^c^	25.38 ± 0.06^b^	0.59 ± 0.08^a^	32.63 ± 0.13^cde^	36.72 ± 0.18^f^	54.3 ± 0.55^g^
δ-tocopherol	0.69 ± 0.06^bc^	0.74 ± 0.02^bc^	0.83 ± 0.04^cd^	0.68 ± 0.04^bc^	0.73 ± 0.05^bc^	0.93 ± 0.06^d^	1.26 ± 0.04^e^	0.14 ± 0.02^a^	0.64 ± 0.03^b^	0.92 ± 0.04^d^	1.65 ± 0.01^f^
PC-8	5.36 ± 0.04^d^	5.37 ± 0.03^d^	3.60 ± 0.08^a^	4.79 ± 0.19^c^	3.73 ± 0.23^a^	4.41 ± 0.02^bc^	4.00 ± 0.07^ab^	n/d	4.33 ± 0.06^b^	3.79 ± 0.09^a^	n/d
α-tocotrienol	n/d	n/d	n/d	n/d	n/d	0.04 ± 0.01^a^	0.15 ± 0.00^b^	0.33 ± 0.01^c^	n/d	n/d	n/d
β-tocotrienol	n/d	n/d	n/d	n/d	n/d	0.10 ± 0.01^a^	0.08 ± 0.00^a^	0.89 ± 0.09^b^	n/d	n/d	n/d
γ-tocotrienol	n/d	n/d	n/d	n/d	n/d	0.15 ± 0.00^a^	0.72 ± 0.01^b^	3.07 ± 0.08^c^	n/d	n/d	n/d
δ-tocotrienol	n/d	n/d	n/d	n/d	n/d	n/d	0.08 ± 0.00^a^	0.86 ± 0.02^b^	n/d	n/d	n/d
total tocochromanols	70.45 ± 0.13^g^	69.24 ± 1.39^fg^	53.82 ± 0.33^a^	65.56 ± 0.43^de^	63.8 ± 0.78^cd^	66.65 ± 0.77^ef^	68.75 ± 0.37^fg^	57.92 ± 0.88^b^	63.16 ± 0.36^cd^	62.24 ± 0.13^c^	57.61 ± 0.54^b^
chlorophyll [mg pheophytin/kg oil]											
	n/d	n/d	0.06 ± 0.01^b^	0.33 ± 0.00^d^	1.07 ± 0.00^e^	0.04 ± 0.00^a^	0.27 ± 0.00^c^	1.32 ± 0.01^f^	n/d	n/d	n/d
DPPH [μmol TE/100 g]											
	168.41 ± 4.82^a^	752.99 ± 5.98^h^	297.89 ± 9.51^e^	313.78 ± 6.51^e^	332.35 ± 6.68^f^	192.69 ± 6.59^b^	214.61 ± 1.49^c^	435.41 ± 6.12^g^	172.89 ± 2.63^a^	219.7 ± 6.68^c^	278.38 ± 7.86^d^
ABTS [μmol TE/100 g]											
	812.94 ± 11.19^a^	1248.50 ± 8.23^d^	812.01 ± 5.24^a^	804.77 ± 4.84^a^	796.84 ± 6.93^a^	890.74 ± 18.8^b^	925.79 ± 10.54^b^	1268.65 ± 16.81^d^	908.03 ± 32.27^b^	984.56 ± 19.07^c^	1023.48 ± 21.06^c^

PC-8—plastochromanol-8; DPPH—2,2-diphenyl-1-picrylhydrazyl; ABTS—2,2′-azino-bis(3-ethylbenzothiazoline-6-sulphonic acid); n/d—not detected; RefO—refined rapeseed oil; rTBHQ—refined rapeseed oil with the addition of tetrabutylhydroquinone; RO5%—a blend of refined rapeseed oil and 5% cold-pressed rapeseed oil; RO25%—a blend of refined rapeseed oil and 25% cold-pressed rapeseed oil; RO100%—cold-pressed rapeseed oil; CO5%—a blend of refined rapeseed oil and 5% cold-pressed coriander seed oil; CO25%—a blend of refined rapeseed oil and 25% cold-pressed coriander seed oil; CO100%—cold-pressed coriander seed oil; AO5%—a blend of refined rapeseed oil and 5% cold-pressed apricot kernel oil; AO25%—a blend of refined rapeseed oil and 25% cold-pressed apricot kernel oil; AO100%—cold-pressed apricot kernel oil. Values of phytosterols, tocochromanols and chlorophyll content are the means of two determinations ± SD. Values of DPPH and ABTS are the means of three determinations ± SD. Means in the same row followed by different letters indicate significant differences (*p* < 0.05) between oil samples.

**Table 2:** Pearson correlation coefficients (r) between antioxidant capacities determined by DPPH and ABTS methods and content of individual homologue tocopherols, total tocochromanols and total phytosterols.

	DPPH	ABTS
α-tocopherol	0.7245	0.8476
β-tocopherol	0.4020	0.5133
γ-tocopherol	0.5458	0.6926
δ-tocopherol	0.2247	0.4159
PC-8	0.5833	0.5782
Total tocochromanols	0.7552	0.9093
Total phytosterols	−0.1273	−0.3488

Significance at *p* < 0.05.

**Table 3:** Component loadings of the quality indices PCA.

Variable	PC1	PC2	PC3
C16:0	−0.7559	−0.4262	0.2409
C18:0	−0.8934	−0.2173	−0.3367
C18:1	−0.9167	−0.3026	0.1927
C18:2	0.7914	0.2835	0.5002
C18:3	0.6932	0.1765	−0.6910
Sum of other acids	−0.4089	0.1144	−0.7154
α-tocopherol	−0.5987	0.7125	−0.2692
β-tocopherol	−0.9657	0.0448	−0.1595
γ-tocopherol	0.1084	0.8915	0.3734
δ-tocopherol	0.0864	0.5501	0.6092
PC-8	0.1510	0.9037	−0.3260
Total tocochromanols	−0.3203	0.9350	0.0190
Total phytosterols	0.6622	0.1642	−0.6838
DPPH	−0.4048	0.7376	−0.0849
ABTS	−0.5191	0.7995	0.1657

## Data Availability

The original contributions presented in the study are included in the article; further inquiries can be directed to the corresponding author.

## Supplementary Materials

The following supporting information can be downloaded at: https://www.mdpi.com/article/10.3390/foods13152336/s1; Table S1. Fatty acid profile of non-heated and heated blends and cold-pressed oils; Table S2: Content of tocochromanols and individual homologues in cold-pressed oils, their unheated and heated blends.

## References

[1] Kalogeropoulos N. (2010). Recovery and Distribution of Macro- and Selected Microconstituents after Pan-frying of Vegetables in Virgin Olive Oil. Olives and Olive Oil in Health and Disease Prevention.

[2] Hazer B. (2023). Macro peroxide initiators based on autoxidized unsaturated plant oils: Block/graft copolymer conjugates for nanotechnology and biomedical applications. JAOCS J. Am. Oil Chem. Soc..

[3] Dodoo D., Adjei F., Tulashie S.K., Adukpoh K.E., Agbolegbe R.K., Gawou K., Manu G.P. (2022). Quality evaluation of different repeatedly heated vegetable oils for deep-frying of yam fries. Meas. Food.

[4] Sun Y., Liu B., Snetselaar L.G., Robinson J.G., Wallace R.B., Peterson L.L., Bao W. (2019). Association of fried food consumption with all cause, cardiovascular, and cancer mortality: Prospective cohort study. BMJ.

[5] Guallar-Castillón P., Rodríguez-Artalejo F., Lopez-Garcia E., León-Muñoz L.M., Amiano P., Ardanaz E., Arriola L., Barricarte A., Buckland G., Chirlaque M.D. (2012). Consumption of fried foods and risk of coronary heart disease: Spanish cohort of the European Prospective Investigation into Cancer and Nutrition study. BMJ.

[6] Cahill L.E., Pan A., Chiuve S.E., Sun Q., Willett W.C., Hu F.B., Rimm E.B. (2014). Fried-food consumption and risk of type 2 diabetes and coronary artery disease: A prospective study in 2 cohorts of US women and men. Am. J. Clin. Nutr..

[7] Shahidi F., Ambigaipalan P., Apak R., Capanoglu E., Shahidi F. (2018). Antioxidants in oxidation control. Measurement of Antioxidant Activity and Capacity: Recent Trends and Applications.

[8] Makahleh A., Saad B., Bari M.F., Shahidi F. (2015). Synthetic phenolics as antioxidants for food preservation. Handbook of Antioxidants for Food Preservation.

[9] Lidon F., Silva M. (2016). An Overview on Applications and Side Effects of Antioxidant Food Additives. Emir. J. Food Agric..

[10] Aydeniz B., Yilmaz E. (2012). Enrichment of frying oils with plant phenolic extracts to extend the usage life. Eur. J. Lipid Sci. Technol..

[11] Kmiecik D., Gramza-Michałowska A., Korczak J. (2018). Anti-polymerization activity of tea and fruits extracts during rapeseed oil heating. Food Chem..

[12] Gangopadhyay N., Lafarga T., Bobo G., Aguiló-Aguayo I. (2021). Oils Extracted from Nuts and Grains. Oil and Oilseed Processing.

[13] Birringer M., Siems K., Maxones A., Frank J., Lorkowski S. (2018). Natural 6-hydroxy-chromanols and -chromenols: Structural diversity, biosynthetic pathways and health implications†. RSC Adv..

[14] Nattagh-Eshtivani E., Barghchi H., Pahlavani N., Barati M., Amiri Y., Fadel A., Khosravi M., Talebi S., Arzhang P., Ziaei R. (2022). Biological and pharmacological effects and nutritional impact of phytosterols: A comprehensive review. Phyther. Res..

[15] Siger A., Dwiecki K., Bąkowska E. (2021). Tocochromanols. Analytical Methods in the Determination of Bioactive Compounds and Elements in Food.

[16] Feng S., Belwal T., Li L., Limwachiranon J., Liu X., Luo Z. (2020). Phytosterols and their derivatives: Potential health-promoting uses against lipid metabolism and associated diseases, mechanism, and safety issues. Compr. Rev. Food Sci. Food Saf..

[17] Winkler J.K., Warner K. (2008). The effect of phytosterol concentration on oxidative stability and thermal polymerization of heated oils. Eur. J. Lipid Sci. Technol..

[18] Nyström L., Achrenius T., Lampi A.M., Moreau R.A., Piironen V. (2007). A comparison of the antioxidant properties of steryl ferulates with tocopherol at high temperatures. Food Chem..

[19] Barrera-Arellano D., Ruiz-Méndez V., Velasco J., Márquez-Ruiz G., Dobarganes C. (2002). Loss of tocopherols and formation of degradation compounds at frying temperatures in oils differing in degree of unsaturation and natural antioxidant content. J. Sci. Food Agric..

[20] Fedko M., Kmiecik D., Siger A., Majcher M. (2022). The Stability of Refined Rapeseed Oil Fortified by Cold-Pressed and Essential Black Cumin Oils under a Heating Treatment. Molecules.

[21] Kmiecik D., Fedko M., Siger A., Kowalczewski P.Ł. (2022). Nutritional Quality and Oxidative Stability during Thermal Processing of Cold-Pressed Oil Blends with 5:1 Ratio of ω6/ω3 Fatty Acids. Foods.

[22] Yang R., Zhang L., Li P., Yu L., Mao J., Wang X., Zhang Q. (2018). A review of chemical composition and nutritional properties of minor vegetable oils in China. Trends Food Sci. Technol..

[23] Liu X., Wang S., Masui E., Tamogami S., Chen J., Zhang H. (2020). Model for prediction of the carbonyl value of frying oil from the initial composition. Lwt.

[24] Grajzer M., Prescha A., Korzonek K., Wojakowska A., Dziadas M., Kulma A., Grajeta H. (2015). Characteristics of rose hip (*Rosa canina* L.) cold-pressed oil and its oxidative stability studied by the differential scanning calorimetry method. Food Chem..

[25] Marmesat S., Morales A., Velasco J., Carmen Dobarganes M. (2012). Influence of fatty acid composition on chemical changes in blends of sunflower oils during thermoxidation and frying. Food Chem..

[26] Liu X., Hoshino N., Wang S., Masui E., Chen J., Zhang H. (2018). A Novel Evaluation Index for Predicting the Degradation Rate of Frying Oils Based on Their Fatty Acid Composition. Eur. J. Lipid Sci. Technol..

[27] Moreno J.J., Mitjavila M.T. (2003). The degree of unsaturation of dietary fatty acids and the development of atherosclerosis (Review). J. Nutr. Biochem..

[28] Joris P.J., Mensink R.P. (2016). Role of cis-Monounsaturated Fatty Acids in the Prevention of Coronary Heart Disease. Curr. Atheroscler. Rep..

[29] Wu H., Xu L., Ballantyne C.M. (2020). Dietary and pharmacological fatty acids and cardiovascular health. J. Clin. Endocrinol. Metab..

[30] Kozłowska M., Gruczyńska E., Ścibisz I., Rudzińska M. (2016). Fatty acids and sterols composition, and antioxidant activity of oils extracted from plant seeds. Food Chem..

[31] Turan S., Topcu A., Karabulut I., Vural H., Hayaloglu A.A. (2007). Fatty acid, triacylglycerol, phytosterol, and tocopherol variations in kernel oil of Malatya apricots from Turkey. J. Agric. Food Chem..

[32] Kostadinović Veličkovska S., Brühl L., Mitrev S., Mirhosseini H., Matthäus B. (2015). Quality evaluation of cold-pressed edible oils from Macedonia. Eur. J. Lipid Sci. Technol..

[33] Sriti J., Aidi W., Thierry W., Mhamdi B., Cerny M., Marzouk B. (2010). Lipid Profiles of Tunisian Coriander (*Coriandrum sativum*) Seed. J. Am. Oil Chem. Soc..

[34] McDowell D., Elliott C.T., Koidis A. (2017). Characterization and comparison of UK, Irish, and French cold pressed rapeseed oils with refined rapeseed oils and extra virgin olive oils. Eur. J. Lipid Sci. Technol..

[35] Packer L., Weber S.U., Rimbach G. (2001). Molecular aspects of α-tocotrienol antioxidant action and cell signalling. J. Nutr..

[36] Schaffer S., Müller W.E., Eckert G.P. (2005). Tocotrienols: Constitutional effects in aging and disease. J. Nutr..

[37] Batool Z., Xu D., Zhang X., Li X., Li Y., Chen Z., Li B., Li L. (2021). A review on furan: Formation, analysis, occurrence, carcinogenicity, genotoxicity and reduction methods. Crit. Rev. Food Sci. Nutr..

[38] Guo Q., Gao S., Sun Y., Gao Y., Wang X., Zhang Z. (2016). Antioxidant efficacy of rosemary ethanol extract in palm oil during frying and accelerated storage. Ind. Crops Prod..

[39] Multari S., Marsol-Vall A., Heponiemi P., Suomela J.P., Yang B. (2019). Changes in the volatile profile, fatty acid composition and other markers of lipid oxidation of six different vegetable oils during short-term deep-frying. Food Res. Int..

[40] Baj A., Cedrowski J., Olchowik-Grabarek E., Ratkiewicz A., Witkowski S. (2019). Synthesis, DFT calculations, and in vitro antioxidant study on novel carba-analogs of vitamin E. Antioxidants.

[41] Seppanen C.M., Song Q., Saari Csallany A. (2010). The antioxidant functions of tocopherol and tocotrienol homologues in oils, fats, and food systems. JAOCS J. Am. Oil Chem. Soc..

[42] Athanasiadis V., Chatzimitakos T., Kalompatsios D., Palaiogiannis D., Makrygiannis I., Bozinou E., Lalas S.I. (2023). Evaluation of the Efficacy and Synergistic Effect of α- and δ-Tocopherol as Natural Antioxidants in the Stabilization of Sunflower Oil and Olive Pomace Oil during Storage Conditions. Int. J. Mol. Sci..

[43] Wagner K.H., Wotruba F., Elmadfa I. (2001). Antioxidative potential of tocotrienols and tocopherols in coconut fat at different oxidation temperatures. Eur. J. Lipid Sci. Technol..

[44] Müller L., Theile K., Böhm V. (2010). In vitro antioxidant activity of tocopherols and tocotrienols and comparison of vitamin E concentration and lipophilic antioxidant capacity in human plasma. Mol. Nutr. Food Res..

[45] White P.J., Artrong L.S. (1986). Effect of selected oat sterols on the deterioration of heated soybean oil. J. Am. Oil Chem. Soc..

[46] Gordon M.H., Magos P. (1983). The effect of sterols on the oxidation of edible oils. Food Chem..

[47] Tolve R., Condelli N., Can A., Tchuenbou-Magaia F.L. (2018). Development and Characterization of Phytosterol-Enriched Oil Microcapsules for Foodstuff Application. Food Bioprocess Technol..

[48] Chang M., Xu Y., Li X., Shi F., Liu R., Jin Q., Wang X. (2020). Effects of stigmasterol on the thermal stability of soybean oil during heating. Eur. Food Res. Technol..

[49] Gibis M., Kruwinnus M., Weiss J. (2015). Impact of different pan-frying conditions on the formation of heterocyclic aromatic amines and sensory quality in fried bacon. FOOD Chem..

[50] Ramadan M.F. (2015). Oxidation of β-sitosterol and campesterol in sunflower oil upon deep- and pan-frying of French fries. J. Food Sci. Technol..

[51] Kreps F., Burčová Z., Schmidt Š. (2017). Degradation of fatty acids and tocopherols to form tocopheryl quinone as risk factor during microwave heating, pan-frying and deep-fat frying. Eur. J. Lipid Sci. Technol..

[52] Hosseini H., Ghorbani M., Meshginfar N., Mahoonak A.S. (2016). A Review on Frying: Procedure, Fat, Deterioration Progress and Health Hazards. JAOCS J. Am. Oil Chem. Soc..

[53] AOCS (2005). Official Method Ce 1h-05. Determination of cis-, trans-, Saturated, Monounsaturated and Polyunsaturated Fatty Acids in Vegetable or Non-ruminant Animal Oils and Fats by Capillary GLC Method. Official and Recommended Practices of the American Oil Chemists’ Society.

[54] Kmiecik D., Fedko M., Rudzińska M., Siger A., Gramza-Michałowska A., Kobus-Cisowska J. (2020). Thermo-Oxidation of Phytosterol Molecules in Rapeseed Oil during Heating: The Impact of Unsaturation Level of the Oil. Foods.

[55] Siger A., Michalak M. (2016). The long-term storage of cold-pressed oil from roasted rapeseed: Effects on antioxidant activity and levels of canolol and tocopherols. Eur. J. Lipid Sci. Technol..

[56] AOCS (2001). Official Method Cc 13i-96 Determination of chlorophyll Pigments in Crude Vegetable Oils. Official Methods and Recommended Practices of the American Oil Chemists’ Society.

[57] Szydłowska-Czerniak A., Tułodziecka A., Momot M., Stawicka B. (2019). Physicochemical, Antioxidative, and Sensory Properties of Refined Rapeseed Oils. JAOCS J. Am. Oil Chem. Soc..

[58] Uitterhaegen E., Sampaio K.A., Delbeke E.I.P., De Greyt W., Cerny M., Evon P., Merah O., Talou T., Stevens C.V. (2016). Characterization of French coriander oil as source of petroselinic acid. Molecules.

[59] Beyzi E., Karaman K., Gunes A., Buyukkilic Beyzi S. (2017). Change in some biochemical and bioactive properties and essential oil composition of coriander seed (*Coriandrum sativum* L.) varieties from Turkey. Ind. Crops Prod..

[60] Symoniuk E., Ksibi N., Wroniak M., Lefek M., Ratusz K. (2022). Oxidative Stability Analysis of Selected Oils from Unconventional Raw Materials Using Rancimat Apparatus. Appl. Sci..

[61] Hassanien M.M.M., Abdel-Razek A.G., Rudzińska M., Siger A., Ratusz K., Przybylski R. (2014). Phytochemical contents and oxidative stability of oils from non-traditional sources. Eur. J. Lipid Sci. Technol..

[62] Ying Q., Wojciechowska P., Siger A., Kaczmarek A., Rudzińska M. (2018). Phytochemical Content, Oxidative Stability, and Nutritional Properties of Unconventional Cold-pressed Edible Oils. J. Food Nutr. Res..

[63] Ramadan M.F., Kroh L.W., Mörsel J.T. (2003). Radical Scavenging Activity of Black Cumin (*Nigella sativa* L.), Coriander (*Coriandrum sativum* L.), and Niger (*Guizotia abyssinica* Cass.) Crude Seed Oils and Oil Fractions. J. Agric. Food Chem..

[64] Lin Y., Knol D., Valk I., van Andel V., Friedrichs S., Lütjohann D., Hrncirik K., Trautwein E.A. (2017). Thermal stability of plant sterols and formation of their oxidation products in vegetable oils and margarines upon controlled heating. Chem. Phys. Lipids.

[65] Soupas L., Huikko L., Lampi A.-m., Piironen V. (2007). Pan-frying may induce phytosterol oxidation. Food Chem..

[66] Chen J., Li D., Tang G., Zhou J., Liu W., Bi Y. (2020). Thermal-Oxidation Stability of Soybean Germ Phytosterols in Different Lipid Matrixes. Molecules.

[67] Salta F.N., Kalogeropoulos N., Karavanou K., Andrikopoulos N.K. (2008). Distribution and retention of phytosterols in frying oils and fried potatoes during repeated deep and pan frying. Eur. Food Res. Technol..

[68] Kmiecik D., Korczak J., Rudzińska M., Gramza-Michałowska A., Hęś M., Kobus-Cisowska J. (2015). Stabilisation of phytosterols by natural and synthetic antioxidants in high temperature conditions. Food Chem..

[69] Zou M., Chen Y., Hu C., He D., Gao P. (2022). Physicochemical properties of rice bran blended oil in deep frying by principal component analysis. J. Food Sci. Technol..

[70] Padovan A., Moret S., Bortolomeazzi R., Moret E., Conchione C., Conte L.S., Brühl L. (2020). Formation of Alkylbenzenes and Tocochromanols Degradation in Sunflower Oil and in Fried Potatoes during Deep-Frying and Pan-Frying. Eur. J. Lipid Sci. Technol..

[71] Cui N., Wang G., Ma Q., Zhao T., Li R., Liang L. (2020). Effect of cold-pressed on fatty acid profile, bioactive compounds and oil oxidation of hazelnut during oxidation process. Lwt.

[72] Winkler-Moser J.K., Bakota E.L., Hwang H.S. (2018). Stability and Antioxidant Activity of Annatto (*Bixa orellana* L.) Tocotrienols During Frying and in Fried Tortilla Chips. J. Food Sci..

[73] Rodríguez G., Squeo G., Estivi L., Quezada Berru S., Buleje D., Caponio F., Brandolini A., Hidalgo A. (2021). Changes in stability, tocopherols, fatty acids and antioxidant capacity of sacha inchi (*Plukenetia volubilis*) oil during French fries deep-frying. Food Chem..

[74] Xu Z., Ye Z., Li Y., Li J., Liu Y. (2020). Comparative study of the oxidation stability of high oleic oils and palm oil during thermal treatment. J. Oleo Sci..

[75] Gumus Z.P., Ustun Argon Z., Celenk V.U., Timur S. (2020). Cold Pressed Tomato (Lycopersicon esculentum L.) Seed Oil.

[76] Mishra S.K., Belur P.D., Iyyaswami R. (2021). Use of antioxidants for enhancing oxidative stability of bulk edible oils: A review. Int. J. Food Sci. Technol..

[77] Yang M., Zheng C., Zhou Q., Huang F., Liu C., Wang H. (2013). Minor components and oxidative stability of cold-pressed oil from rapeseed cultivars in China. J. Food Compos. Anal..

[78] Chew S.C. (2020). Cold pressed rapeseed (*Brassica napus*) oil. Cold Pressed Oils.

[79] Dordevic D., Dordevic S., Ćavar-Zeljković S., Kulawik P., Kushkevych I., Tremlová B., Kalová V. (2022). Monitoring the quality of fortified cold-pressed rapeseed oil in different storage conditions. Eur. Food Res. Technol..

[80] Floegel A., Kim D.O., Chung S.J., Koo S.I., Chun O.K. (2011). Comparison of ABTS/DPPH assays to measure antioxidant capacity in popular antioxidant-rich US foods. J. Food Compos. Anal..

[81] Shang W., Dong H., Strappe P., Zhou Z., Blanchard C. (2018). Characterization of endogenous antioxidant attributes and its influence on thermal stability of canola oil. RSC Adv..

[82] Kobyliński J.P., Krygier K., Karlovits G., Szydłowska-Czerniak A. (2016). Effect of Specific Oil Surface Area on the Thermal Stressing of Rapeseed Oil during Heating in an Electric Frying Pan. JAOCS J. Am. Oil Chem. Soc..

[83] Giuffrè A.M., Zappia C., Capocasale M. (2017). Effects of High Temperatures and Duration of Heating on Olive Oil Properties for Food Use and Biodiesel Production. JAOCS J. Am. Oil Chem. Soc..

[84] Yu K.S., Cho H., Hwang K.T. (2018). Physicochemical properties and oxidative stability of frying oils during repeated frying of potato chips. Food Sci. Biotechnol..

[85] Farooq S., Abdullah, Zhang H., Weiss J. (2021). A comprehensive review on polarity, partitioning, and interactions of phenolic antioxidants at oil–water interface of food emulsions. Compr. Rev. Food Sci. Food Saf..

[86] Wang Z., Zhang Y., Tu Z., Yu C., Liu R., Deng Z., Luo T. (2024). The degradation and antioxidant capacity of anthocyanins from eggplant peels in the context of complex food system under thermal processing. Food Biosci..

[87] Ioannou E.T., Gliatis K.S., Zoidis E., Georgiou C.A. (2023). Olive Oil Benefits from Sesame Oil Blending While Extra Virgin Olive Oil Resists Oxidation during Deep Frying. Molecules.

[88] Kmiecik D., Fedko M., Siger A., Kulczyński B. (2019). Degradation of tocopherol molecules and its impact on the polymerization of triacylglycerols during heat treatment of oil. Molecules.

[89] Liu R., Xu Y., Chang M., Tang L., Lu M., Liu R., Jin Q., Wang X. (2021). Antioxidant interaction of α-tocopherol, γ-oryzanol and phytosterol in rice bran oil. Food Chem..

[90] Tang L., Liu R., Xu Y., Zhang X., Liu R., Chang M., Wang X. (2022). Synergistic and antagonistic interactions of α-tocopherol, γ-oryzanol and phytosterol in refined coconut oil. Lwt.

[91] Ramírez-Anaya J.d.P., Castañeda-Saucedo M.C., Olalla-Herrera M., Villalón-Mir M., Serrana H.L.-G.d.l., Samaniego-Sánchez C. (2019). Changes in the antioxidant properties of extra virgin olive oil after cooking typical mediterranean vegetables. Antioxidants.

