# Supplementary material for: The Effect of High-Temperature Heating on Amounts of Bioactive Compounds and Antiradical Properties of Refined Rapeseed Oil Blended with Rapeseed, Coriander and Apricot Cold-Pressed Oils
Source: Foods. 2024 Jul 25;13(15):2336. doi: 10.3390/foods13152336 (PMC11311388; doi:10.3390/foods13152336)
Supplement: Supplementary file 1 [file foods-13-02336-s001.zip › Table S1-fatty acids.pdf]

Table S1. Fatty acids profile at not heated and heated blends and cold-pressed oils

		RefO	rTBHQ	RO5%	RO25%	RO100%	CO5%	CO25%	CO100%	AO5%	AO25%	AO100%
**C16:0**	not heated	4.21±0.03^{cAB}	4.20±0.04^{bAB}	4.15±0.02^{cA}	4.20±000^{dAB}	4.14±0.02^{bA}	4.16±0.04^{cA}	4.32±0.03^{cBC}	4.72±0.00^{dD}	4.11±0.00^{cA}	4.24±0.03^{cAB}	4.43±0.01^{dC}
	170°C	4.22±0.02^{cA} (0.01)	4.33±0.16^{bA} (0.13)	4.20±0.02^{cA} (0.05)	4.23±0.01^{dA} (0.03)	-	4.19±0.01^{cA} (0.03)	4.35±0.01^{cA} (0.03)	-	4.15±0.02^{cA} (0.04)	4.25±0.01^{cA} (0.01)	-
	200°C	4.32±0.03^{dBC} (0.11)	4.31±0.01^{bBC} (0.11)	4.30±0.02^{dB} (0.15)	4.36±0.01^{eCD} (0.16)	-	4.29±0.01^{dAB} (0.13)	4.44±0.01^{dE} (0.12)	-	4.24±0.01^{dA} (0.13)	4.38±0.01^{dD} (0.14)	-
**C18:0**	not heated	1.40±0.01^{bBC}	1.42±0.02^{aBC}	1.47±0.01^{bC}	1.50±0.00^{abcCD}	1.67±0.02^{aE}	1.60±0.01^{bDE}	1.90±0.01^{bF}	3.21±0.04^{cG}	1.47±0.01^{bC}	1.35±0.01^{bB}	1.06±0.00^{cA}
	170°C	1.41±0.01^{bB} (0.01)	1.43±0.03^{aB} (0.01)	1.48±0.01^{bC} (0.01)	1.50±0.01^{bcC} (0.00)	-	1.61±0.01^{bD} (0.01)	1.92±0.01^{bE} (0.02)	-	1.50±0.01^{bC} (0.03)	1.35±0.01^{bA} (0.00)	-
	200°C	1.43±0.01^{bB} (0.03)	1.44±0.01^{aB} (0.02)	1.52±0.01^{bC} (0.05)	1.54±0.01^{cC} (0.04)	-	1.65±0.00^{bD} (0.05)	1.93±0.01^{bE} (0.03)	-	1.51±0.00^{bC} (0.04)	1.38±0.01^{bA} (0.03)	-
**C18:1**	not heated	65.36±0.03^{kA}	65.34±0.03^{fA}	65.86±0.02^{kB}	65.56±0.01^{lAB}	66.61±0.05^{eC}	67.60±0.05^{kE}	69.71±0.05^{kG}	82.18±0.09^{fI}	67.12±0.08^{kD}	67.97±0.06^{kF}	70.22±0.01^{fH}
	170°C	65.78±0.03^{lB} (0.42)	65.45±0.19^{fA} (0.11)	66.15±0.06^{lC} (0.29)	65.83±0.05^{mB} (0.27)	-	67.90±0.02^{lE} (0.30)	69.97±0.05^{lG} (0.26)	-	67.49±0.06^{lD} (0.37)	68.3±0.06^{lF} (0.33)	-
	200°C	66.44±0.05^{mAB} (1.08)	66.26±0.10^{gA} (0.92)	66.95±0.05^{mC} (1.09)	66.58±0.06^{nB} (1.02)	-	68.60±0.03^{mE} (1.00)	70.74±0.05^{mG} (1.03)	-	68.21±0.03^{mD} (1.09)	68.98±0.04^{mF} (1.01)	-
**C18:2**	not heated	18.68±0.05^{jF}	18.63±0.05^{deF}	18.37±0.00^{jE}	18.21±0.02^{jE}	16.72±0.02^{dC}	17.53±0.01^{jD}	15.96±0.01^{jB}	7.83±0.02^{eA}	18.33±0.03^{jE}	19.34±0.00^{jG}	23.33±0.02^{eH}
	170°C	18.47±0.03^{iD} (-0.21)	18.92±0.24^{eE} (0.29)	18.20±0.02^{iCD} (-0.17)	18.08±0.03^{jC} (-0.13)	-	17.36±0.01^{iB} (-0.17)	15.78±0.02^{iA} (-0.18)	-	18.09±0.02^{iC} (-0.24)	19.14±0.02^{iE} (-0.20)	-
	200°C	18.08±0.05^{hE} (-0.60)	18.17±0.05^{dE} (-0.46)	17.71±0.02^{hD} (-0.66)	17.58±0.03^{iC} (-0.63)	-	16.91±0.02^{hB} (-0.62)	15.31±0.02^{hA} (-0.65)	-	17.66±0.02^{hCD} (-0.67)	18.62±0.01^{hF} (-0.72)	-
**C18:3**	not heated	9.10±0.02^{gG}	9.08±0.01^{cG}	8.83±0.01^{gF}	9.11±0.01^{hG}	9.13±0.00^{cG}	7.84±0.01^{gE}	6.80±0.00^{gD}	0.33±0.01^{aB}	7.85±0.04^{gE}	6.32±0.01^{gC}	0.07±0.00^{aA}
	170°C	8.89±0.02^{fC} (-0.21)	8.44±0.76^{cBC} (-0.64)	8.65±0.02^{fC} (-0.18)	8.94±0.02^{gC} (-0.17)	-	7.67±0.01^{fB} (-0.17)	6.66±0.02^{fA} (-0.14)	-	7.65±0.03^{fB} (-0.20)	6.16±0.02^{fA} (-0.16)	-
	200°C	8.49±0.02^{eE} (-0.61)	8.57±0.04^{cE} (-0.51)	8.19±0.01^{eD} (-0.64)	8.5±0.04^{fE} (-0.61)	-	7.30±0.02^{eC} (-0.54)	6.28±0.02^{eB} (-0.52)	-	7.27±0.01^{eC} (-0.58)	5.85±0.01^{eA} (-0.47)	-
**other**	not heated	1.25±0.01^{aD}	1.34±0.01^{aE}	1.31±0.01^{aE}	1.42±0.00^{abF}	1.73±0.00^{aG}	1.26±0.01^{aD}	1.31±0.00^{aE}	1.72±0.02^{bG}	1.11±0.00^{aC}	0.78±0.00^{aA}	0.90±0.00^{bB}
	170°C	1.23±0.02^{aBC} (-0.02)	1.43±0.15^{aD} (0.09)	1.32±0.02^{aBCD} (0.01)	1.42±0.01^{aCD} (0.00)	-	1.27±0.01^{aBCD} (0.01)	1.32±0.01^{aBCD} (0.01)	-	1.12±0.02^{aB} (0.01)	0.79±0.01^{aA} (0.01)	-
	200°C	1.25±0.02^{aC} (0.00)	1.25±0.01^{aC} (-0.09)	1.33±0.01^{aD} (0.02)	1.44±0.02^{abE} (0.02)	-	1.25±0.01^{aC} (-0.01)	1.29±0.02^{aCD} (-0.02)	-	1.11±0.01^{aB} (0.00)	0.8±0.01^{aA} (0.02)	-

RefO – refined rapeseed oil; rTBHQ – refined rapeseed oil with the addition of tetrabutylhydroquinone; RO5% – a blend of refined rapeseed oil and 5% cold-pressed rapeseed oil; RO25% – a blend of refined rapeseed oil and 25% cold-pressed rapeseed oil; RO100% – cold-pressed rapeseed oil; CO5% – a blend of refined rapeseed oil and 5% cold-pressed coriander seed oil; CO25% – a blend of refined rapeseed oil and 25% cold-pressed coriander seed oil; CO100% – cold-pressed coriander seed oil; AO5% – a blend of refined rapeseed oil and 5% cold-pressed apricot kernel oil; AO25% – a blend of refined rapeseed oil and 25% cold-pressed apricot kernel oil; AO100% – cold-pressed apricot kernel oil; other – C14:0, C16:1, C20:0, C20:1, C22:0, C22:1. Values for samples of unheated blends and cold-pressed oils are the means of two determinations ± SD. Values for heated samples are the means of four determinations ± SD. Means in the same column followed by different lower case letters indicate significant differences ($p<0.05$) between samples heated at different temperature and between fatty acids. Means in the same row followed by different capital letters indicate significant differences ($p<0.05$) between oil samples. The increase (+) or decrease (-) share of individual fatty acids was marked in brackets.
