# Supplementary material for: The Effect of High-Temperature Heating on Amounts of Bioactive Compounds and Antiradical Properties of Refined Rapeseed Oil Blended with Rapeseed, Coriander and Apricot Cold-Pressed Oils
Source: Foods. 2024 Jul 25;13(15):2336. doi: 10.3390/foods13152336 (PMC11311388; doi:10.3390/foods13152336)
Supplement: Supplementary file 1 [file foods-13-02336-s001.zip › Table S2-tocochromanols.pdf]

Table S2. Content of tocochromanols and individual homologues in cold-pressed oils and their unheated and heated blends [mg/100 g of oil]

		RefO	rTBHQ	RO5%	RO25%	RO100%	CO5%	CO25%	CO100%	AO5%	AO25%	AO100%
**α-tocopherol**	not heated	30.70±0.05^{iE}	30.18±0.48^{fE}	25.20±0.23^{jC}	28.18±0.11^{gD}	26.41±0.24^{cC}	29.60±0.45^{hE}	36.66±0.48^{iF}	50.68±0.49^{dG}	25.45±0.12^{gC}	20.76±0.28^{fB}	1.66±0.00^{aA}
	170°C	1.62±0.12^{bcAB}	29.01±0.35^{fD}	2.00±0.09^{cB}	5.6±0.36^{cdC}	-	2.09±0.13^{cdB}	4.69±1.20^{eC}	-	1.13±0.35^{abAB}	0.73±0.23^{abA}	-
	200°C	0.40±0.11^{aC}	0.87±0.02^{abD}	0.22±0.08^{abAB}	0.29±0.08^{aBC}	-	n/d	0.15±0.03^{abA}	-	n/d	n/d	-
**β-tocopherol**	not heated	0.13±0.03^{aAB}	0.16±0.02^{abB}	0.05±0.01^{abAB}	0.15±0.05^{aAB}	0.09±0.01^{aAB}	0.19±0.01^{abcB}	0.43±0.05^{abC}	1.39±0.08^{bD}	0.11±0.03^{abAB}	0.06±0.01^{abAB}	n/d
	170°C	0.06±0.04^{aAB}	0.13±0.02^{aC}	0.04±0.02^{aA}	0.03±0.02^{aA}	-	0.11±0.00^{abBC}	0.27±0.03^{abD}	-	0.03±0.02^{abA}	0.03±0.03^{aA}	-
	200°C	0.03±0.01^{aAB}	0.05±0.03^{aB}	n/d	n/d	-	n/d	0.09±0.03^{aC}	-	n/d	n/d	-
**γ-tocopherol**	not heated	33.58±0.09^{jE}	32.78±0.95^{gDE}	24.15±0.16^{iB}	31.78±0.26^{hCD}	32.84±0.25^{dDE}	31.23±0.22^{hC}	25.38±0.06^{hB}	0.59±0.08^{abA}	32.63±0.13^{hCDE}	36.72±0.18^{gF}	54.3±0.55^{bG}
	170°C	15.17±0.60^{gABC}	32.15±0.28^{gD}	15.16±0.08^{gABC}	17.37±0.24^{eC}	-	15.93±2.01^{fBC}	13.53±0.96^{fAB}	-	12.63±1.10^{eA}	13.43±1.97^{dAB}	-
	200°C	8.09±0.63^{eD}	10.01±0.22^{dE}	5.77±0.44^{eC}	4.94±0.86^{cdC}	-	3.27±0.20^{deB}	2.51±0.14^{cdAB}	-	2.92±0.41^{cdB}	1.73±0.07^{abcA}	-
**∂-tocopherol**	not heated	0.69±0.06^{abBC}	0.74±0.02^{abBC}	0.83±0.04^{bCD}	0.68±0.04a^{BC}	0.73±0.05^{aBC}	0.93±0.06^{abcD}	1.26±0.04^{abcE}	0.14±0.02^{abA}	0.64±0.03^{abB}	0.92±0.04^{abcD}	1.65±0.01^{aF}
	170°C	0.58±0.04^{aAB}	0.68±0.03^{abBCD}	0.57±0.07^{abAB}	0.64±0.04^{aABC}	-	0.72±0.07^{abcCD}	1.1±0.01^{abcE}	-	0.54±0.05^{abA}	0.81±0.11^{abD}	-
	200°C	0.43±0.02^{aA}	0.43±0.05^{abA}	0.35±0.03^{abA}	0.34±0.04^{aA}	-	0.40±0.05^{abcA}	0.65±0.04^{abB}	-	0.34±0.07^{abA}	0.42±0.02^{abA}	-
**PC-8**	not heated	5.36±0.04^{dE}	5.37±0.03^{cE}	3.60±0.08^{dB}	4.79±0.19^{cdD}	3.73±0.23^{bB}	4.41±0.02^{eCD}	4.00±0.07^{deBC}	n/d	4.33±0.06^{dC}	3.79±0.09^{cB}	n/d
	170°C	2.03±0.08^{cCD}	5.12±0.12^{cF}	2.17±0.11^{cDE}	2.42±0.09^{bE}	-	1.86±0.27^{bcdCD}	1.77±0.17^{bcBC}	-	1.47±0.1^{bcAB}	1.22±0.16^{abcA}	-
	200°C	0.9±0.10^{abD}	1.13±0.10^{bE}	0.60±0.05^{abC}	0.52±0.09^{aC}	-	0.26±0.02^{abB}	0.26±0.04^{abAB}	-	0.28±0.05^{abB}	0.11±0.01^{abA}	-
**α-tocotrienol**	not heated	n/d	n/d	n/d	n/d	n/d	0.04±0.01^{abcA}	0.15±0.00^{abB}	0.33±0.01^{abC}	n/d	n/d	n/d
	170°C	n/d	n/d	n/d	n/d	n/d	0.03±0.01^{aA}	0.03±0.01^{aA}	-	n/d	n/d	-
	200°C	n/d	n/d	n/d	n/d	n/d	n/d	n/d	-	n/d	n/d	-
**β-tocotrienol**	not heated	n/d	n/d	n/d	n/d	n/d	0.10±0.01^{abcA}	0.08±0.00^{abA}	0.89±0.09^{abB}	n/d	n/d	n/d
	170°C	n/d	n/d	n/d	n/d	n/d	0.03±0.00^{aA}	0.04±0.01^{aA}	-	n/d	n/d	-
	200°C	n/d	n/d	n/d	n/d	n/d	n/d	n/d	-	n/d	n/d	-
**γ-tocotrienol**	not heated	n/d	n/d	n/d	n/d	n/d	0.15±0.00^{abcA}	0.72±0.01^{abcB}	3.07±0.08^{cC}	n/d	n/d	n/d
	170°C	n/d	n/d	n/d	n/d	n/d	0.04±0.01^{abA}	0.36±0.06^{abB}	-	n/d	n/d	-
	200°C	n/d	n/d	n/d	n/d	n/d	n/d	0.08±0.00^{a}	-	n/d	n/d	-
**∂-tocotrienol**	not heated	n/d	n/d	n/d	n/d	n/d	n/d	0.08±0.00^{abA}	0.86±0.02^{abB}	n/d	n/d	n/d
	170°C	n/d	n/d	n/d	n/d	n/d	n/d	0.06±0.01^{a}	-	n/d	n/d	-
	200°C	n/d	n/d	n/d	n/d	n/d	n/d	0.05±0.00^{a}	-	n/d	n/d	-
**total tocochromanols**	not heated	70.45±0.13^{kG}	69.24±1.39^{iFG}	53.82±0.33^{kA}	65.56±0.43^{iDE}	63.8±0.78^{eCD}	66.65±0.77^{iEF}	68.75±0.37^{jFG}	57.92±0.88^{eB}	63.16±0.36^{iCD}	62.24±0.13^{hC}	57.61±0.54^{cB}
	170°C	19.45±0.78^{hABC}	67.09±0.74^{hE}	19.93±0.32^{hBC}	26.06±0.61^{fD}	-	20.81±2.38^{gC}	21.85±2.40^{gC}	-	15.80±1.56^{fA}	16.22±2.4^{eAB}	-
	200°C	9.85±0.85^{fD}	12.49±0.33^{eE}	6.94±0.58^{fC}	6.08±1.03^{dC}	-	3.93±0.17^{eB}	3.8±0.21^{deB}	-	3.54±0.52^{dAB}	2.25±0.05^{bcA}	-

PC-8 – plastochromanol-8; n/d – not detected; RefO – refined rapeseed oil; rTBHQ – refined rapeseed oil with the addition of tetrabutylhydroquinone; RO5% – a blend of refined rapeseed oil and 5% cold-pressed rapeseed oil; RO25% – a blend of refined rapeseed oil and 25% cold-pressed rapeseed oil; RO100% – cold-pressed rapeseed oil; CO5% – a blend of refined rapeseed oil and 5% cold-pressed coriander seed oil; CO25% – a blend of refined rapeseed oil and 25% cold-pressed coriander seed oil; CO100% – cold-pressed coriander seed oil; AO5% – a blend of refined rapeseed oil and 5% cold-pressed apricot kernel oil; AO25% – a blend of refined rapeseed oil and 25% cold-pressed apricot kernel oil; AO100% – cold-pressed apricot kernel oil. Values for samples of unheated blends and cold-pressed oils are the means of two determinations ± SD. Values for heated samples are the means of four determinations ± SD. Means in the same column followed by different lower case letters indicate significant differences ($p<0.05$) between samples heated at different temperature and between homologues tocochromanols. Means in the same row followed by different capital letters indicate significant differences ($p<0.05$) between oil samples.
